# Assessment of pain due to lumbar spine diseases using MR spectroscopy: a preliminary report

**DOI:** 10.1007/s00776-013-0357-6

**Published:** 2013-02-27

**Authors:** Shoji Yabuki, Shin-ichi Konno, Shin-ichi Kikuchi

**Affiliations:** Department of Orthopaedic Surgery, Fukushima Medical University School of Medicine, 1 Hikarigaoka, Fukushima, Fukushima 960-1295 Japan

## Abstract

**Background data:**

There is a considerable difference in pain perception among individuals. In patients with chronic pain, recent studies using fMRI, PET and SPECT have shown that functional changes mainly occurred in the anterior cingulate cortex (ACC), prefrontal cortex (PFC) and thalamus. Brain magnetic resonance spectroscopy (MRS) can evaluate brain chemistry by measuring metabolites such as *N*-acetyl aspartate (NAA). The purpose of this study was to analyze whether brain MRS could assess pain due to lumbar spine diseases.

**Methods:**

NAA levels were determined relative to the concentration of creatine/phosphocreatine complex (Cr) and choline (Cho), which is commonly used as an internal standard. The NAA/Cr and NAA/Cho ratios in the ACC, PFC and thalamus were compared between six patients with unilateral pain (left side) and six control patients without pain.

**Results:**

In the right thalamus (contralateral side to symptom), the NAA/Cr in the patients with pain was statistically significantly lower compared with the control patients (*p* < 0.05). Also, in the right thalamus, the NAA/Cho in pain patients was significantly lower compared with controls (*p* < 0.01). When considering just the right thalamus, there were statistically significant correlations between the numerical rating scale for pain (NRS) and NAA values.

**Conclusions:**

Lumbar pain can be assessed indirectly by analyzing the decrease in NAA concentration in the thalamus.

## Introduction

Pain is one of the most frequent symptoms in lumbar spine diseases, as evaluated using a numerical rating scale (NRS), visual analog scale (VAS) and/or faces pain scale [1, 2]. However, there is a considerable difference in pain perception among individuals. Patients with lumbar spine diseases sometimes complain of severe pain that cannot be explained by physical findings or imaging studies. If pain is measured objectively, the pathogenesis of lumbar spine diseases and/or therapeutic efficacy may be evaluated more accurately. Thus, when considering that the pain pathway for objective pain measurement is ultimately recognized in the brain [3], cerebral imaging and/or metabolic studies can be useful.

Recent brain imaging such as functional MRI (fMRI) showed morphological and functional changes in the brain of patients with chronic pain [4–8]. Single-voxel proton magnetic resonance spectroscopy (MRS) is a non-invasive examination determining the cell metabolism of tissues and organs. A number of studies indicate that MRS can detect biochemical changes associated with functional brain abnormalities, such as epilepsy [9], dementia, Parkinson’s disease, schizophrenia and depression [10]. Grachev and colleagues [11] have reported that in chronic low back pain (CLBP) patients, reductions in *N*-acetyl aspartate (NAA) and glucose were observed in the prefrontal cortex (PFC). Recently, Sharma and colleagues [12] showed that NAA levels in the primary somatosensory cortex decreased in patients with CLBP. Also, Gussew and colleagues [13] demonstrated that reductions in NAA were observed in the anterior insula and anterior cingulated cortex in patients with non-specific CLBP.

CLBP pain is the most common cause of employees missing work for a long period [14]. It has been reported that CLBP is closely associated with depressive and anxiety states [15], and long-term LBP further exacerbates such psychiatric conditions [16]. When evaluating pain (LBP and sciatica) due to lumbar spine diseases using MRS, patients with a shorter duration of pain and without severe psychiatric conditions may be good candidates for analysis. The purpose of this study was to analyze whether MRS in these patients could assess pain due to lumbar spine diseases.

## Subjects and methods

This study was approved by our institutional review board (no. 1254), and informed consent was obtained from each subject and control. Subjects studied included six patients complaining of unilateral pain (left side) due to lumbar spine diseases. The numerical rating scale (NRS) showed symptom severity was most painful during the day because pain became worse when moving and walking. Subject gender consisted of two males and four females. Age ranged from 28 to 68 years old (mean age 40 years). Diseases included two with disc herniation, three with spinal stenosis and one with idiopathic low back pain. Symptom duration was from 2 to 12 months (mean duration 5.7 months). Six healthy subjects without pain were used for control (Table 1). There were no significant differences in gender and age between the patient and the control groups. The brief scale for psychiatric problems in orthopedic patients (BS-POP) for medical personnel was used for evaluating psychiatric states. Verification of reliability, validity and reproducibility of the BS-POP has already been confirmed [17].

**Table 1:** Summary of subjects

Subjects or control	Age	Gender	Diagnosis	Symptom	NRS of pain	Duration of pain (months)	BS-POP
Subject 1	68	Female	LCS	Lt. sciatica	7	12	8
Subject 2	38	Female	LDH	Lt. LBP and sciatica	6	2	10
Subject 3	38	Female	LCS	Lt. sciatica	3	8	9
Subject 4	34	Male	LDH	Lt. sciatica	7	3	10
Subject 5	28	Male	Discopathy	Lt. LBP	8	2	11
Subject 6	34	Female	LCS	Lt. LBP and sciatica	4	7	8
Control 1	52	Male	–	–	0	–	–
Control 2	56	Male	–	–	0	–	–
Control 3	24	Male	–	–	0	–	–
Control 4	23	Female	–	–	0	–	–
Control 5	27	Male	–	–	0	–	–
Control 6	69	Female	–	–	0	–	–

*LCS* lumbar canal stenosis, *LDH* lumbar disc herniation, *LBP* low back pain, *NRS* numerical rating scale for pain, *BS-POP* brief scale for psychiatric problems in orthopedic patients

**Fig. 1:**
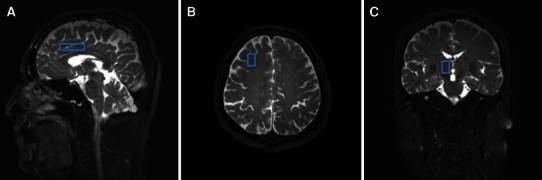
Location of the spectroscopic voxel in the brain. **a** Anterior cingulated cortex (ACC). **b** Prefrontal cortex (PFC). **c** Thalamus

**Fig. 2:**
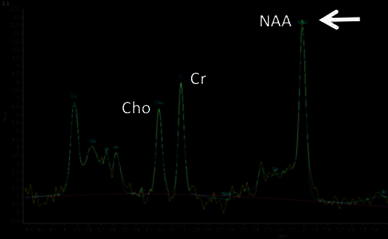
Proton localized spectra. *NAA* *N*-acetyl aspartate, *Cr* creatine/phosphocreatine complex, *Cho* choline

All MRI and MRS studies were performed with a 3-T clinical imaging instrument (Achieva 3.0T, Philips, The Netherlands). High-resolution sagittal and axial views were used for identification of the anterior cingulated cortex (ACC), prefrontal cortex (PFC) and thalamus (Fig. 1). Proton localized spectra were collected using point-resolved spectroscopy (PRESS). The settings for taking MRS were TR 2000 ms, TE 36 ms, voxel size 20 mm × 15 mm × 15 mm and NSA [number of sample (signals) averaged] 128. In the current study, we focused on *N*-acetyl aspartate (NAA) (Fig. 2). The value of NAA was measured relative to the concentration of the creatine/phosphocreatine complex (Cr) and choline (Cho), which is commonly used as an internal standard [4]. The NAA/Cr and NAA/Cho ratios in the ACC, PFC and thalamus were compared between the subjects and the controls. In all subjects, MRS was taken before treatment with medication or neuronal blocks.

### Statistical analysis

Data were expressed as the mean ± SD. A non-parametric test (Mann-Whitney *U* test) was used for comparison among groups. Pearson’s correlation coefficients were used to analyze the correlations between NRS and NAA values. *p* values <0.05 were considered statistically significant difference.

## Results

On the psychiatric states evaluated by the BS-POP, five of six patients had normal scores, with one patient having an abnormal borderline score (11 points). Therefore, we concluded that these patients had no severe psychiatric problems.

On the NAA/Cr, there were no statistically significant differences between the two groups in the bilateral ACC, PFC and left thalamus. However, in the right thalamus (contralateral side to the symptom), the NAA/Cr in the subjects (1.29 ± 0.62) was statistically significantly lower compared with controls (1.54 ± 0.17; *p* < 0.05) (Table 2). On the NAA/Cho, there were also no statistically significant differences between the two groups in the bilateral ACC, PFC and left thalamus. However, in the right thalamus (contralateral side to the symptom), the NAA/Cho in the subjects (1.59 ± 0.097) was statistically significantly lower compared with controls (1.92 ± 0.16; *p* < 0.005) (Table 3). All NAA/Cr and NAA/Cho data for the thalamus are shown in Table 4. When the differences between the right and left side of the NAA/Cr and NAA/Cho in the thalamus were compared between the subjects and the control group, only the NAA/Cr ratio was statistically significant different (*p* < 0.05).

**Table 2:** NAA/Cr compared between subjects and control

	Subjects (mean ± SD)	Control (mean ± SD)	*p* value
R ACC	1.313 ± 0.189	1.242 ± 0.134	0.337
L ACC	1.169 ± 0.478	1.355 + 0.077	0.63
R PFC	1.609 ± 0.252	1.651 ± 0.136	0.873
L PFC	1.526 ± 0.154	1.560 ± 0.136	0.688
R thalamus	1.292 ± 0.062	1.536 ± 0.172	0.025
L thalamus	1.328 ± 0.103	1.439 ± 0.141	0.15

*R* right, *L* left, *ACC* anterior circulated cortex, *PFC* prefrontal cortex

**Table 3:** NAA/Cho compared between subjects and control

	Subjects (mean ± SD)	Control (mean ± SD)	*p* value
R ACC	1.473 ± 0.244	1.476 ± 0.224	1
L ACC	1.277 ± 0.548	1.497 ± 0.178	0.631
R PFC	2.488 ± 0.465	2.498 ± 0.238	0.522
L PFC	2.274 ± 0.548	2.218 ± 0.264	1
R thalamus	1.586 ± 0.097	1.919 ± 0.163	0.006
L thalamus	1.738 ± 0.239	1.783 ± 0.161	0.81

*R* right, *L* left, *ACC* anterior cingulated cortex, *PFC* prefrontal cortex

**Table 4:** NAA/Cr and NAA/Cho in the thalamus

Subjects or control	NAA/Cr in the rt. thalamus	NAA/Cr in the lt. thalamus	NAA/Cho in the rt. thalamus	NAA/Cho in the lt. thalamus
Subject 1	1.34	1.25	1.6	2.03
Subject 2	1.336	1.301	1.721	1.779
Subject 3	1.322	1.231	1.639	1.437
Subject 4	1.19	1.42	1.52	1.79
Subject 5	1.24	1.49	1.44	1.92
Subject 6	1.323	1.278	1.596	1.471
Control 1	1.67	1.35	2.02	1.92
Control 2	1.3	1.27	1.65	1.59
Control 3	1.51	1.46	1.99	1.98
Control 4	1.79	1.66	2.11	1.67
Control 5	1.46	1.53	1.92	1.87
Control 6	1.484	1.364	1.826	1.666

**Fig. 3:**
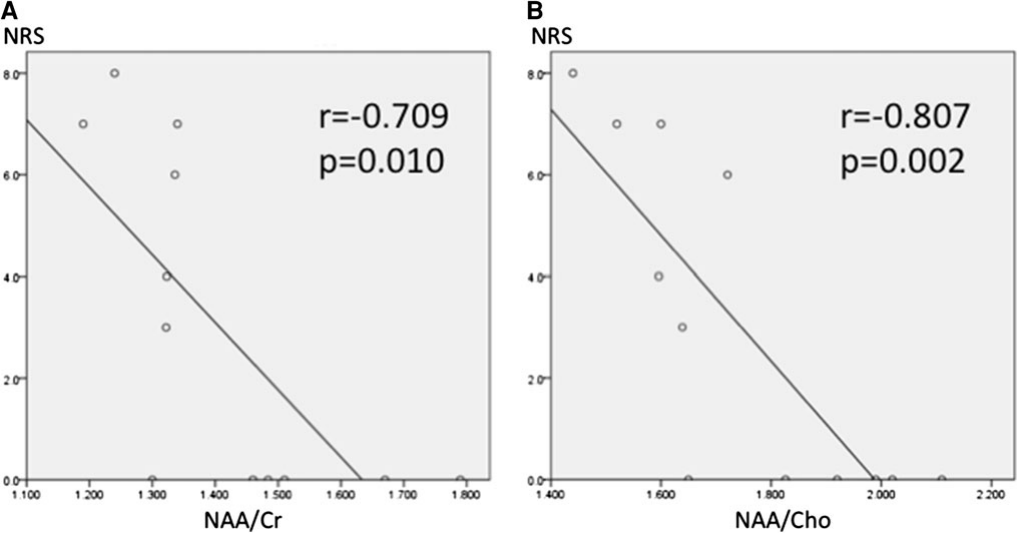
Correlation between the NRS and NAA in the thalamus. **a** Correlation between the NRS and NAA/Cr. **b** Correlation between the NRS and NAA/Cho. *NRS* Numerical rating scale for pain, *NAA* *N*-acetyl aspartate, *Cr* creatine/phosphocreatine complex, *Cho* choline

When focused on the right thalamus, there were statistically significant correlations between the NRS and NAA values (Fig. 3). In the ACC and PFC, there were no significant correlations between the NRS and NAA values.

## Discussion

This study demonstrated that the patients with unilateral pain (LBP and/or sciatica) due to lumbar spine diseases with shorter duration (within 12 months) of pain and without severe psychiatric conditions showed decreases in the NAA/Cr and NAA/Cho in the thalamus on the contralateral side from the symptom. There were statistically significant correlations between NRS and NAA values. Lumbar pain can possibly be assessed indirectly by analyzing the decrease in the NAA concentration in the thalamus.

Recent pain studies using fMRI [6–8], positron emission tomography (PET) [18, 19] and single-photon emission computed tomography (SPECT) [20, 21] have shown that functional changes mainly occurred in the ACC, PFC and thalamus in patients with chronic pain. PET and fMRI studies in the healthy subjects demonstrated that acute pain activated the primary and secondary somatosensory cortex (S1 and S2), insular cortex, ACC, PFC and thalamus [22]. These areas and the basal ganglia, cerebellum, amygdala, hippocampus and regions within the parietal and temporal cortices are often called the “pain matrix.” The matrix can be thought of as having lateral components (sensory-discriminatory, involving areas such as the primary and secondary somatosensory cortices, thalamus and posterior parts of the insula) and medial components (affective-cognitive-evaluative, involving areas such as the anterior parts of the insula, ACC and PFC) [23]. The current study showed NAA reduction only in the contralateral thalamus. This finding may suggest that the subjects in this study had little affective-cognitive-evaluative factor associated with their pain. However, the “pain matrix” is not a definitive entity because different brain regions play a more or less active role depending on the precise interplay of the factors involved in influencing pain perception, for example, cognition and mood.

In the current study, we used MRS for evaluation of lumbar pain (LBP and sciatica). An exact correlation between MRS and other brain imaging is still uncertain [22]. The NAA is localized within neurons and involved in synaptic processes. It has been observed to decrease in various conditions involving neuronal cell damage and loss and is therefore thought to be a neuronal and axonal marker [11, 22, 24]. CLBP is a multifactorial, pathological condition involving psychiatric problems, which may influence the results of NAA concentration [25]. In the current study, pain duration was less than 12 months, and the BS-POP was essentially normal. Therefore, the results of this study may show pain due to lumbar spine diseases without influence of psychiatric problems.

There is some variability in MRS results. It has been reported that the NAA concentration and NAA/Cho were higher in the left thalamus by 21.9 and 20 %, respectively, in healthy subjects [26]. However, the results of the current study showed lower NAA/Cr and NAA/Cho in the left thalamus in the control group (Table 4). The cause is not clear. Side matching in the same subjects may be important for proton MRS studies. All of the subjects in the current preliminary report had left side pain. Regarding the relationship between age and NAA, Charles et al. [27] reported that the choline, creatine and NAA were lower in older subjects in the voxel representing cortical and subcortical gray matter. Recent studies by Grachev et al. [28, 29] demonstrated that there was no evidence for NAA correlation strength differences in the thalamus, insula, orbital frontal cortex and sensorimotor cortex between the young-aged group and middle-aged group. Regarding the relationship between gender and NAA, Charles et al. [27] and Nagae-Poetscher et al. [26] reported that there were no differences between males and females. Therefore, age matching may be more important than gender matching for comparative studies of disease states using proton MRS.

**Fig. 4:**
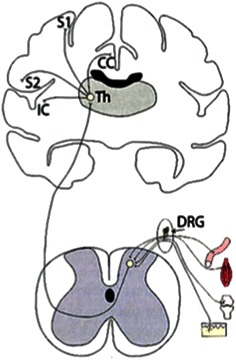
Pain pathway. Every sensory system except for the olfactory system includes a thalamic nucleus that receives sensory signals and sends them to the associated primary cortical area. *S1* Primary somatosensory cortex, *S2* secondary somatosensory cortex, *IC* insular cortex (reproduced from [24] with permission)

The thalamus plays an important role in the pain pathway. Every sensory system except for the olfactory system includes a thalamic nucleus that receives sensory signals and sends them to the associated primary cortical area (Fig. 4) [30]. The relation between NAA concentration in the thalamus and pain has been previously reported. Pattany and colleagues [31] showed that the NAA concentration was negatively correlated with pain intensity in patients with chronic neuropathic pain after spinal cord injury. Fukui and colleagues [32] showed a decrease in the NAA concentration on the contralateral side in seven of nine neuropathic pain patients. Our study showed similar results using subjects with a shorter duration of pain. The NAA concentration in the thalamus may decrease during the acute stage of pain. However, the relationship among NAA changes, duration of pain and therapeutic efficacy was not clarified in the current study. A decrease in thalamic perfusion has been reported in patients with chronic pain using SPECT [20, 21]. If effective treatment is not given and the NAA reduction lasts for a long time, a chronic pain condition may develop in patients with severe acute pain. Further studies are needed to clarify these important points.

The current study has several limitations. The number of patients was very small. Strict age matching and gender matching were not performed. The symptom duration was short. The MRI instrument had no linear combination model (LC model) software that could measure the absolute concentration of NAA [33, 34]. We analyzed the NAA concentration in only three brain regions (ACC, PFC and thalamus). Further studies in more patients with various pain conditions are needed. In conclusion, lumbar pain in patients with shorter durations of pain and without severe psychiatric conditions may be objectively assessed indirectly by analyzing the decrease in the NAA concentration in the thalamus.
